# The Association Between Dietary Inflammatory Potential and Sex Hormones in Male Children and Adolescents Aged 6–19 Years

**DOI:** 10.3389/fendo.2021.722941

**Published:** 2021-08-03

**Authors:** Zheng Qin, Nuozhou Liu, Ruoxi Liao, Luojia Jiang, Baihai Su

**Affiliations:** ^1^Department of Nephrology, National Clinical Research Center for Geriatrics, West China Hospital, Sichuan University, Chengdu, China; ^2^Med+ Biomaterial Institute of West China Hospital/West China School of Medicine of Sichuan University, Chengdu, China; ^3^Med-X Center for Materials, Sichuan University, Chengdu, China; ^4^West China School of Medicine, West China Hospital of Sichuan University, Chengdu, China

**Keywords:** dietary inflammatory index, testosterone, estradiol, sex hormone, NHANES, cross-sectional study

## Abstract

**Aims:**

This study aimed to assess the relationship between dietary inflammatory index (DII) and sex hormones in male children and adolescents aged 6-19 years.

**Methods:**

We obtained data from the 2013-2016 National Health and Nutrition Examination Survey (NHANES). Male participants aged 6-19 years old with the complete data of DII and sex hormones were included. Weighted multiple regression analysis and subgroup analysis were preformed to estimate the independent relationship between DII and sex hormones.

**Results:**

A total of 1717 male participants with the average age of 13.02 ± 3.82 years were enrolled, of whom 41.3% (n=713) were children and 58.47% (n=1004) were adolescents. In children, mean DII was 0.18 ± 1.67, with scores ranging from -4.53 to 4.08. As for adolescents, the mean DII was 0.36 ± 1.98, mean total testosterone (TT) was 376.94 ± 206.69 ng/dl overall. A negative association between DII with TT and estradiol (E2) was observed (TT: β=-11.97, P=0.0006; E2: β=-0.45, P=0.0108) in male adolescent. Subgroup analysis and interaction test results indicated that this association was similar in male adolescents with different body mass index. No statistically significant association was observed in children.

**Conclusions:**

Pro-inflammatory diet was associated with lower TT and E2 level in male adolescent, while no association with statistical significance between them was observed in male children. However, more studies are still needed to validate the causal relationship between DII and sex hormones.

## Introduction

Testosterone is an essential sex hormone produced by Leydig cell in testis and regulated by negative feedback of hypothalamic-pituitary-gonadal axis (HPGA) (1). Once testosterone enters the circulation, almost 50% of it is tightly bound to sex hormone-binding globulin (SHBG, a circulating homodimeric glycoprotein mainly synthesized in liver) and become unavailable for biological functions (2). About 2% of testosterone remains unbound and called free testosterone (FT), while the rest of it (approximately 48%) is bound to albumin with low affinity compared with SHBG-bound testosterone. Only FT and albumin-bound testosterone can diffuse into target cell membrane and play its physiological function. FT, albumin-bound testosterone and SHBG-bound testosterone are jointly called total testosterone (TT) (3). One quarter to half of circulating estradiol (E2) is estimated to derive from highly active aromatase in Leydig cell, which transferring testosterone into E2, while the rest of it deriving from peripheral aromatization of testosterone, especially in adipose tissue, muscle, bone and brain (4). Previous studies have demonstrated that TT, SHBG and E2 not only play essential roles in reproduction, such as onset of puberty and the subsequent sexual maturation, including spermatogenesis and secondary sex characteristic formation and maintenance, *etc.*, but also non-sexual biological process, including bone growth, body composition, and the metabolism of glucose, lipid and protein (4–6). A lack of sex steroid may lead to delay of puberty, reduced bone density and size, obesity, impaired insulin sensitivity, and increased risk of dyslipidemia, hypertension, metabolic syndrome and cardiovascular diseases (6–9). Accumulative studies have suggested that sex hormones play a key role in the development and growth in children and adolescents and the disorder of sex hormones may result in a heavy disease burden (10). Thus, the management of sex hormones in children and adolescents is of great significance.

Dietary Inflammatory Index (DII), a literature-derived dietary tool, could measure individual dietary inflammatory potential (11). Higher DII score indicates a more pro-inflammatory diet by increased inflammatory markers, such as interleukin-6 (IL-6) and C-reactive protein (CRP), and lower DII score indicates a more anti-inflammatory diet (12, 13). DII has also been served as a marker for glycaemia, including HbA1c, insulin resistance (IR) and post-load glucose (14). Previous studies have reported that a greater DII score was positively associated with higher cancer incidence and mortality, increased risk of depression symptoms, cardiovascular disease and its motility, *etc.* (15–19).

The relationship between inflammation and sex hormones still remains unclear. Several cross-sectional human studies revealed an inverse relationship between testosterone and inflammatory markers including IL-6, TNF-α and so on (20–22). Moreover, high levels of endogenous inflammatory cytokines in hypogonadal men can be reversed by testosterone replacement, indicating that inflammation may contribute to reduced testosterone level (23). The supplementation of E2 could also attenuate inflammation by partially inhibiting neutrophil infiltration (24). As for animal experiments, Ajithkumar et al. found sex hormones in mice regulates visceral adipose tissue inflammation *via* shaping the transcriptional landscape of Treg cells in a BLIMP1 transcription factor-dependent manner (25). Aged rats treated with etanercept (a TNF-α inhibitor) showed obviously higher inflammatory markers while decreased serum testosterone (26). In induced colitis mouse model, E2 could inhibit inflammatory cytokines to produce an anti-inflammatory effect through the estrogen receptor β signaling pathway (27). It could be inferred that the consumption of inflammatory diet may also have impact on the sex hormones. Zhang et al. reported that although there was no association between DII and sex hormones with statistical significance, male adults with a more pro-inflammatory diet appeared to have a higher risk of testosterone deficiency (28). However, the association between DII and sex hormones in male children and adolescents has not been reported before.

Therefore, using data from the National Health and Nutrition Examination Survey (NHANES), the aim of this study was to explore the association of dietary inflammatory potential and sex hormones in male children and adolescents, which may shed new light on the management of sex hormone in clinical practice.

## Materials and Methods

### Study Population

Data in this study was obtained from NHANES, a cross-sectional study in order to investigate the nutrition and health status in US population. It was conducted on a repeated two-year cycle by the National Center for Health Statistics (NCHS) (29). All NHANES data are publicly available at https://www.cdc.gov/nchs/nhanes/.

**Figure 1:**
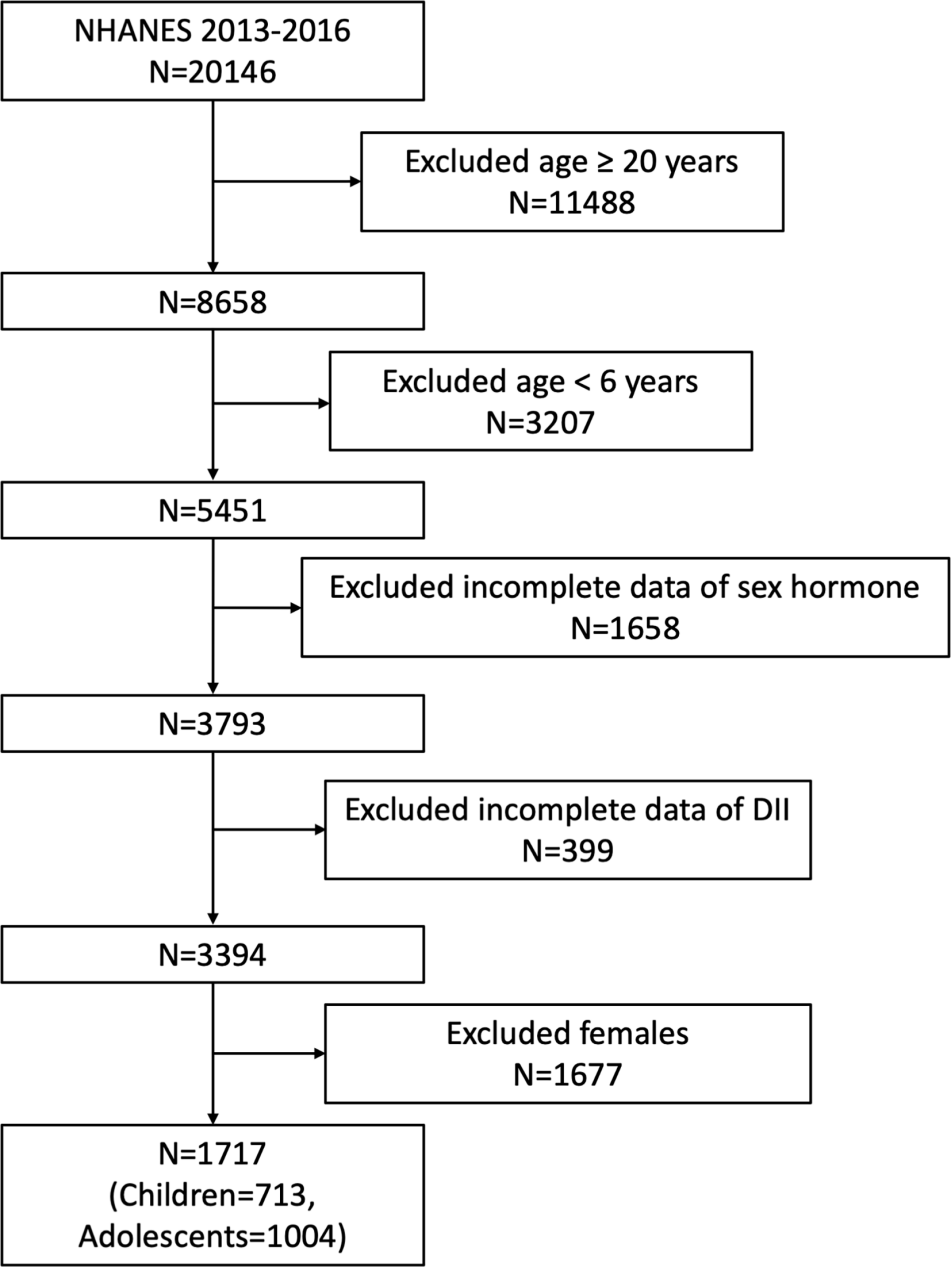
Flowchart of the sample selection from NHANES 2013-2016.

Our study was based on the survey cycles from NHANES 2013-2014 and 2015-2016, since only these two cycles included both data on sex hormone and dietary information to calculate DII. A total of 20146 participants was enrolled at first, after the exclusion of participants aged ≥ 20 years (n=11488), <6 years (n=3207), missing the data about sex hormone (n= 1658), missing the dietary data about DII (n= 399) and female participants (n=1677), 1717 eligible male participants aged 6-19 years were included in our final analysis. Considering the different condition between children and adolescents, we further regarded children and adolescents as those aged 6-11 years (n=713) and 12-19 years (n=1004) according to previous studies and analyzed these two groups independently (5, 30) (**Figure 1**).

The ethics review board of the NCHS approved all NHANES protocols and written informed consent was obtained from all participants or a parent and/or legal guardian for participants aged below 16 years old.

### Exposure and Outcome Definitions

DII was designed as exposure variable. Dietary data in NHANES was obtained by a 24-h dietary recall at the mobile examination center and validated by the Nutrition Methodology Working Group (31, 32). We calculate the DII score based on the 24-h dietary recall data according to the calculating protocol and used this to assess the dietary inflammatory potential for each participant (33). A higher positive DII score suggested a pro-inflammatory effect and a lower negative DII score suggested an anti-inflammatory effect of diet (33). 27 food parameters were available in NHANES and used to calculate DII, including alcohol, β-carotene, cholesterol, carbohydrates, energy, fats, fibers, folic acid, iron, magnesium, zinc, selenium, vitamin A, vitamin B-6, vitamin B-12, vitamin C, vitamin D, vitamin E, mono-unsaturated fatty acid, protein, niacin, riboflavin, n-3 fatty acid, n-6 fatty acid, poly-unsaturated fatty acid, saturated fat and thiamin. Previous studies have demonstrated that using less than 30 food parameters to calculate DII would not affect its predictive ability (34–36). We analyzed DII as a continuous variable and divided participants into three groups based on DII tertiles to conduct further analysis.

Sex hormones, including total testosterone (TT), estradiol (E2) and SHBG in serum were quantified. TT and E2 were measured *via* isotope dilution liquid chromatography tandem mass spectrometry (ID-LC-MS/MS) method based on the National Institute for Standards and Technology’s (NIST) reference method. The measurement of SHBG was based on the reaction of SHBG with immuno-antibodies and chemo-luminescence measurements of the reaction products that occurs after two incubation periods and subjecting to a magnetic field. The microparticles were captured on an electrode, where a chemiluminescent reaction occurs and could be measured by a photomultiplier tube. We also calculate free androgen index as TT (ng/dl) divided by SHBG (nmol/l) and the ratio of TT to E2, which evaluated the approximate amount of circulating free testosterone and aromatase activity indirectly (37, 38). Outcome variables in this study included TT, E2, SHBG, free androgen index and the ratio of TT to E2.

### Covariates

Covariates in our study included age (year), body mass index (BMI, Kg/m^2^), race, education level, ratio of family income to poverty (RIP), energy intake (kcal), protein intake (g), the time of blood sample collection (time of venipuncture), serum cotinine (ng/mL), hypertension, diabetes and pubertal status. BMI was categorized as <25, 25-29.9 and ≥ 30 kg/m^2^, which corresponded to normal weight, overweight and obese population for participants aged ≥18 years. The same groups were also categorized according to a standard definition for child overweight and obesity reported before for those aged 6-17 years subjects (39). Since sex hormones may have diurnal fluctuations, the time of blood sample collection was treated as a covariate in our analysis and classified as morning, afternoon and evening (40). Serum cotinine level was included to assess the status of tobacco smoke exposure (30). Considering the pubertal status may distort the associations between DII and sex hormones, pubertal condition was also included in our analysis. We defined puberty as TT ≥ 50 ng/dl for males according to previous studies (9, 41, 42). All detailed measurement processes of these variable were publicly available at www.cdc.gov/nchs/nhanes/.

### Statistical Analysis

All statistical analysis was conducted according to CDC guidelines (43). An appropriate NHANES sampling weights was used and accounted for complex multistage cluster survey design in the analysis. Children and adolescents were grouped as those aged 6-11 years (n=713) and 12-19 years (n=1004) and analyzed independently. Continuous variables were presented as mean with standard deviation and categorical variables were presented as percentage. Either a weighted student’s t test (for continuous variables) or weighted chi-square test (for categorical variables) was used to evaluate the differences in groups divided by DII (tertiles). Weighted multivariable linear regression was employed to explore the independent relationship between DII and sex hormones (including TT, E2, SHBG, free androgen index and the ratio of TT to E2) in three different models. In model 1, no covariates were adjusted. Model 2 was adjusted for age and race. Model 3 was adjusted for age, race, education level, PIR, BMI, energy intake, protein intake, time of venipuncture, serum cotinine, hypertension, diabetes and pubertal status. Considering the effect of BMI on sex hormones, subgroup analysis stratified by BMI was further conducted using stratified multivariable linear regression model. In addition, BMI was also treated as a pre-specified potential effect modifier. An interaction term was added to test the heterogeneity of associations between the subgroups. P< 0.05 was considered statistically significant. All analysis was preformed using Empower software (www.empowerstats.com; X&Y solutions, Inc., Boston MA) and R version 3.4.3 (http://www.R-project.org, The R Foundation).

## Results

### Baseline Characteristics of Participants

A total of 1717 male participants with the average age of 13.02 ± 3.82 years and average DII of 0.30 ± 1.88 were enrolled in this study, of whom 41.3% (n=713) were children and 58.47% (n=1004) were adolescents. Children aged 6-11 years trended to have lower DII, TT, E2, free androgen index, ratio of TT to E2 and higher SHBG level than adolescents aged 12-10 years (all P<0.05). 6.42% children and 92.62% reached the puberty, respectively (**Table 1**).

**Table 1:** Baseline characteristics of participants, weighted.

	Overall	Children (aged 6-11 years)	Adolescents (aged 12-19 years)	P value
n (%)	N=1717	713 (41.53)	1004 (58.47)	/
DII	0.30 ± 1.88	0.18 ± 1.67	0.36 ± 1.98	0.0594
Age (year)	13.02 ± 3.82	8.62 ± 1.70	15.37 ± 2.24	<0.0001
Race				
Mexican American	16.26	16.23	16.27	0.2997
Other Hispanic	8.55	10.50	7.51	
Non-Hispanic White	54.14	52.07	55.25	
Non-Hispanic Black	12.58	12.89	12.42	
Other Races	8.47	8.31	8.55	
Educational level				
Less than high school	73.40	99.88	59.24	<0.0001
High school or GED	23.08	0.12	35.42	
Above high school	2.96	0	4.55	
Unknown	0.55	0	0.78	
PIR (%)				
<1	22.50	24.00	21.68	0.2869
≥1	77.50	76.00	78.32	
BMI				
Normal weight	75.62	90.46	67.69	<0.0001
Overweight	13.92	7.96	17.11	
Obese	10.46	1.58	15.20	
Time of venipuncture				
Morning	40.72	33.97	44.33	<0.0001
Afternoon	38.73	41.06	37.48	
Evening	20.55	24.97	18.19	
Puberty (%)	62.59	6.42	92.62	<0.0001
Hypertension (%)^1^	0.85	/	2.77	<0.0001
Diabetes (%)	0.43	0.29	0.51	0.8265
Serum cotinine (ng/mL)	7.57 ± 38.91	0.25 ± 0.84	11.47 ± 47.72	<0.0001
Energy intake (kcal)	2202.71 ± 1005.50	1980.76 ± 711.21	2321.39 ± 1113.89	<0.0001
Protein intake (g)	82.44 ± 51.39	69.95 ± 30.80	89.12 ± 58.47	<0.0001
Total testosterone (ng/dl)	251.61 ± 241.38	17.21 ± 54.92	376.94 ± 206.69	<0.0001
Estradiol (pg/ml)	13.10 ± 11.41	2.47 ± 1.58	18.78 ± 10.28	<0.0001
SHBG (nmol/l)	62.25 ± 46.27	102.86 ± 49.30	40.54 ± 25.15	<0.0001
Free androgen index	8.03 ± 8.28	0.33 ± 1.34	12.15 ± 7.45	<0.0001
Ratio of TT to Estradiol	15.52 ± 13.64	4.35 ± 8.91	21.50 ± 11.86	<0.0001

GED, general educational development; PIR, the ratio of family income to poverty; BMI, body mass index; SHBG, sex hormone-binding globulin; TT, total testosterone.

1 Data of hypertension status for participants aged 6-11 years was not available.

In children, mean DII was 0.18 ± 1.67, with scores ranging from -4.53 (most pro-inflammatory) to 4.08 (anti-inflammatory). The ranges of DII for tertiles 1-3 were -4.53 to -0.57, -0.57 to 1.03, 1.03 to 4.08, respectively. Mean TT was 16.65 ± 54.00, 15.67 ± 50.26 and 19.94 ± 61.45 ng/dl for tertiles 1 to 3, however, there was no statistical significance (P=0.6931). In addition, no significant difference was observed in E2, SHBG, free androgen index and ratio of TT to E2 as well (all P>0.05) (**Supplementary Table 1**).

As for adolescents, the mean DII was 0.36 ± 1.98, and the ranges of DII tertiles 1-3 were -4.53 to -0.51, -0.51 to 1.43 and 1.43 to 4.58. Among three DII tertiles, differences with statistical significance were observed in energy intake, protein intake, serum cotinine and TT (all P<0.05). Mean TT was 376.94 ± 206.69 ng/dl overall, with the average TT of 400.38 ± 222.14 ng/dl for the lowest tertile and 367.68 ± 202.21 ng/dl for the highest tertile (P=0.0414).The difference between tertiles in E2, SHBG, free androgen index and ratio of TT to E2 did not meet the statistical significance (all P>0.05) (**Supplementary Table 1**).

### The Association Between DII and Sex Hormone in Male Children Aged 6–11 Years

Weighted multivariable regression analysis was conducted to estimate the association of DII with sex hormones for children in three different models (**Table 2**). In the fully adjusted model (Model 3), a negative association between DII and TT was observed (β=-2.98, 95%CI: -4.39, 10.36), however, this association did not meet the statistical significance (P=0.4278).

**Table 2:** Association between dietary inflammatory index with sex hormone in children aged 6-11 and adolescents aged 12-19, weighted.

DII	β^1^ (95% CI^2^), P value					
	In children aged 6-11			In adolescents aged 12-19		
	Model 1^3^	Model 2^4^	Model 3^5^	Model 1^3^	Model 2^4^	Model 3^5^
**Total testosterone (ng/dl)**						
Continuous	0.15 (-2.27, 2.57) 0.9035	-0.42 (-2.64, 1.80) 0.7131	-0.78 (-1.12, 2.67) 0.4219	-8.59 (-15.02, -2.15) 0.0090	-12.83 (-18.47, -7.19) <0.0001	-11.97 (-18.77, -5.17) 0.0006
Tertile 1	Reference	Reference	Reference	Reference	Reference	Reference
Tertile 2	-0.85 (-10.65, 8.96) 0.8656	-1.15 (-10.13, 7.83) 0.8021	-0.87 (-5.20, 6.94) 0.7793	-38.82 (-69.93, -7.70) 0.0147	-42.34 (-69.41, -15.28) 0.0022	-41.47 (-65.84, -17.09) 0.0009
Tertile 3	-2.01 (-7.95, 11.97) 0.6921	-2.86 (-12.04, 6.31) 0.5408	-2.98 (-4.39, 10.36) 0.4278	-30.45 (-61.98, 1.09) 0.0587	-46.79 (-74.36, -19.22) 0.0009	-40.77 (-71.07, -10.48) 0.0085
**Estradiol (pg/ml)**						
Continuous	0.01 (-0.06, 0.08) 0.7934	-0.00 (-0.07, 0.06) 0.9061	0.03 (-0.04, 0.10) 0.3789	-0.28 (-0.60, 0.05) 0.0930	-0.52 (-0.77, -0.26) <0.0001	-0.45 (-0.80, -0.11) 0.0108
Tertile 1	Reference	Reference	Reference	Reference	Reference	Reference
Tertile 2	0.05 (-0.23, 0.34) 0.7230	-0.04 (-0.33, 0.21) 0.6643	0.11 (-0.15, 0.38) 0.4007	-0.76 (-2.33, 0.81) 0.3417	-1.79 (-3.04, -0.55) 0.0049	-1.00 (-2.55, -0.55) 0.0263
Tertile 3	0.08 (-0.20, 0.36) 0.5762	0.08 (-0.19, 0.35) 0.5648	0.14 (-0.08, 0.35) 0.2164	-1.75 (-3.30, -0.20) 0.0270	-1.95 (-3.18, -0.73) 0.0018	-1.36 (-2.61, -0.12) 0.0325
**SHBG (nmol/l)**						
Continuous	-0.18 (-2.35, 1.99) 0.8734	0.92 (-1.02, 2.87) 0.3506	-0.31 (-2.50, 1.87) 0.7785	-0.02 (-0.81, 0.76) 0.9532	0.58 (-0.10, 1.25) 0.0940	0.36 (-0.53, 1.26) 0.4270
Tertile 1	Reference	Reference	Reference	Reference	Reference	Reference
Tertile 2	-1.60 (-10.40, 7.20) 0.7219	-1.47 (-9.32, 6.38) 0.7143	-1.82 (-8.83, 5.19) 0.6110	-1.30 (-5.09, 2.50) 0.5032	-0.85 (-4.08, 2.37) 0.6038	-2.46 (-5.67, 0.74) 0.1328
Tertile 3	-2.98 (-11.91, 5.96) 0.5143	2.46 (-5.56, 10.48) 0.5486	-1.63 (-10.13, 6.88) 0.7076	-0.55 (-4.40, 3.30) 0.7802	2.01 (-1.28, 5.29) 0.2316	0.30 (-3.68, 4.28) 0.8827
**Free androgen index**						
Continuous	0.00 (-0.06, 0.06) 0.9501	-0.01 (-0.07, 0.04) 0.6695	0.04 (-0.02, 0.09) 0.1987	-0.17 (-0.41, -0.06) 0.0425	-0.39 (-0.57, -0.22) <0.0001	-0.19 (-0.42, -0.05) 0.0154
Tertile 1	Reference	Reference	Reference	Reference	Reference	Reference
Tertile 2	0.08 (-0.16, 0.31) 0.5306	0.07 (-0.15, 0.29) 0.5316	0.13 (-0.05, 0.30) 0.1541	-0.88 (-2.01, 0.24) 0.1236	-1.06 (-1.91, -0.21) 0.0150	-0.35 (-1.19, 0.49) 0.4112
Tertile 3	0.05 (-0.20, 0.29) 0.7140	-0.07 (-0.29, 0.16) 0.5646	0.15 (-0.06, 0.36) 0.1646	-0.21 (-1.35, 0.92) 0.7123	-1.14 (-2.01, -0.27) 0.0102	0.06 (-0.98, 1.10) 0.9103
**Ratio of TT to Estradiol**						
Continuous	-0.06 (-0.45, 0.33) 0.7613	-0.16 (-0.51, 0.19) 0.3708	0.19 (-0.10, 0.48) 0.2024	-0.19 (-0.56, 0.18) 0.3070	-0.15 (-0.53, 0.22) 0.4234	0.01 (-0.48, 0.49) 0.9723
Tertile 1	Reference	Reference	Reference	Reference	Reference	Reference
Tertile 2	-0.94 (-2.53, 0.65) 0.2455	-1.04 (-2.45, 0.36) 0.1461	-0.48 (-1.41, 0.44) 0.3063	-0.02 (-1.81, 1.77) 0.9814	0.04 (-1.75, 1.84) 0.9635	-0.41 (-2.15, 1.33) 0.6454
Tertile 3	0.25 (-1.36, 1.86) 0.7632	-0.54 (-1.98, 0.90) 0.4629	0.94 (-0.18, 2.07) 0.0993	-0.91 (-2.72, 0.91) 0.3277	-0.66 (-2.49, 1.16) 0.4768	-0.58 (-2.74, 1.59) 0.6009

In sensitivity analysis, dietary inflammatory index was converted from a continuous variable to a categorical variable (tertiles).

1 β: effect sizes.

2 95% CI: 95% confidence interval.

3 Model 1: no covariates were adjusted.

4 Model 2: adjusted for age and race.

5 Model 3: adjusted for age, race, education level, ratio of family income to poverty, body mass index, energy intake, protein intake, time of venipuncture, serum cotinine, hypertension, diabetes and pubertal status.

We further tested this association after DII treated as tertiles, while there was still no significant association between them was found (all P>0.05). As for the association between DII and other sex hormones (including E2, SHBG, free androgen index and ratio of TT to E2), we did not observe any statistically significant association (all P>0.05). Subgroup group stratified by BMI (normal weight, overweight and obese) showed the similar results without statistical significance as well (all P>0.05) (**Supplementary Table 2**).

### The Association Between DII and Sex Hormone in Male Adolescents Aged 12–19 Years

We also employed weighted multivariable linear regression to evaluate this association for male adolescents aged 12-19 years (**Table 2**). Our results revealed a negative association between DII with TT with statistical significance (Model 1, β=-8.59, 95% CI: -15.02, -2.15, P=0.0090; Model 2, β=-12.83, 95% CI: -18.47, -7.19, P<0.0001; Model 3, β=-11.97, 95%CI: -18.77, -5.17, P=0.0006). According to the results of fully adjusted model (Model 3), for each unit of the increased DII score was associated a TT decrease by 11.97 ng/dl, suggesting that the higher DII scores were associated with lower TT level. This association remained statistically significant after DII was grouped as tertiles. The fully adjusted effect size (reference to Tertile 1) was -41.47 for Tertile 2 (95%CI: -65.84, -17.09, P=0.0009) and -40.77 for Tertile 3 (95%CI: -71.07, -10.48, P=0.0085), suggesting that male adolescents in Tertile 2 and Tertile 3 had a mean 41.47 and 40.77 ng/dl decrease in TT compared with those in Tertile 1.

A negative association with statistical significance was also observed between DII and E2 (Model 1, β=-0.28, 95% CI: -0.60, 0.05, P=0.0930; Model 2, β=-0.52, 95% CI: -0.77, -0.26, P<0.0001; Model 3, β=-0.45, 95%CI: -0.80, -0.11, P=0.0108). After full adjustment, each unit of the increased DII score was corresponded to a E2 decrease by 0.45 pg/ml in male adolescents. When DII was treated as tertiles, this association still remained stable. Participants in Tertile 2 and Tertile 3 had a mean 1.00 and 1.36 pg/ml decrease in E2 compared with those in Tertile 1 (Tertile 1: reference; Tertile 2: β=-1.00, 95% CI: -2.55, -0.55, P=0.0263; Tertile 3: β=-1.36, 95% CI: -2.61, -0.12, P=0.0325).

In the full adjusted model, we also found that DII was negatively associated with free androgen index (β=-0.19, 95% CI: -0.42, -0.05, P=0.0154), while this association did not exist when DII was classified as tertiles (all P>0.05). In addition, we did not find any significant relationship between DII with SHBG and the ratio of TT to E2 (all P>0.05).

There was no significant difference was found in the association between DII and sex hormones among different BMI groups (for TT, P for interaction=0.1980; for E2, P for interaction= 0.3388; for E2, P for interaction= 0.5807; for free androgen index, P for interaction= 0.1669; for the ratio of TT to E2, P for interaction= 0.5582), suggesting that there was no significant dependence of BMI on this association. After adjusting for all covariates expect BMI, we observed that higher DII was negatively associated with TT, E2 and free androgen index among overweight and obese participants. For overweight male adolescents, each unit increase of DII was associated with a decrease by 14.86 ng/dl, 1.38 pg/ml and 0.61 in TT, E2 and free androgen index. For obese male adolescents, each unit increase of DII was associated with a decrease by 16.83 ng/dl in TT, 0.33 pg/ml in E2 and 0.22 in free androgen index. When DII was categorized as tertiles, the negative association still remained stable in TT and E2, while it did not meet the statistical significance in free androgen index, which was consistent with the results for the whole sample reported above. Our findings indicated that higher consumption of pro-inflammatory diet was associated with lower TT and E2. However, no association with statistical significance was observed among the male adolescents with normal weight (**Table 3**).

**Table 3:** Subgroup analysis of association between DII and sex hormone stratified by BMI groups in adolescents aged 12-18.

DII Tertile	β^1^ (95% CI^2^), P value				
	Total testosterone (ng/dl)	Estradiol (pg/ml)	SHBG (nmol/l)	Free androgen index	Ratio of TT to Estradiol
Normal weight					
Continuous	1.26 (-15.39, 17.90) 0.8826	-0.45 (-1.56, 0.66) 0.4285	-0.52 (-1.71, 0.67) 0.3961	0.14 (-0.63, 0.91) 0.7213	-0.13 (-1.26, 1.01) 0.8262
Tertile 1	Reference	Reference	Reference	Reference	Reference
Tertile 2	-14.69 (-66.07, 36.68) 0.5761	-2.68 (-6.10, 0.73) 0.1263	-1.63 (-5.34, 2.07) 0.3888	-0.13 (-2.49, 2.23) 0.9168	-0.69 (-4.21, 2.83) 0.7029
Tertile 3	36.74 (-43.47, 116.95) 0.3710	-0.58 (-5.92, 4.75) 0.8302	-3.20 (-8.98, 2.59) 0.2808	3.37 (-0.31, 7.06) 0.0751	1.09 (-4.41, 6.58) 0.6989
Overweight					
Continuous	-14.86 (-30.95, -1.23) 0.0325	-1.38 (-2.53, -0.23) 0.0200	0.05 (-1.32, 1.43) 0.9386	-0.61 (-1.34, -0.11) 0.0466	0.13 (-0.58, 0.84) 0.7258
Tertile 1	Reference	Reference	Reference	Reference	Reference
Tertile 2	-3.32 (-55.24, -61.88) 0.0116	-0.95 (-5.16, -3.26) 0.0388	-1.90 (-6.84, 3.04) 0.4521	-0.83 (-3.45, 1.78) 0.5319	0.64 (-1.90, 3.19) 0.6217
Tertile 3	-12.09 (-62.05, -26.23) 0.0298	-2.09 (-7.42, -3.24) 0.0428	-1.29 (-7.55, 4.96) 0.6859	0.15 (-3.15, 3.46) 0.9281	0.79 (-2.44, 4.01) 0.6322
Obese					
Continuous	-16.83 (-25.62, -8.04) 0.0002	-0.33 (-0.71, -0.05) 0.0201	0.49 (-0.77, 1.75) 0.4462	-0.22 (-0.48, -0.03) 0.0361	-0.03 (-0.72, 0.66) 0.9242
Tertile 1	Reference	Reference	Reference	Reference	Reference
Tertile 2	-62.14 (-93.50, -30.79) 0.0001	-1.51 (-2.87, -0.15) 0.0305	-1.58 (-6.10, 2.93) 0.4922	-0.56 (-1.48, 0.35) 0.2250	-0.57 (-3.04, 1.90) 0.6505
Tertile 3	-72.90 (-111.55, -34.24) 0.0002	-0.95 (-2.63, -0.72) 0.0456	0.71 (-4.85, 6.28) 0.8019	-0.52 (-1.64, 0.60) 0.3639	-1.37 (-4.42, 1.68) 0.3782
P for interaction	0.1076	0.4099	0.5309	0.1637	0.6012

The showing results of subgroup analysis was adjusted for adjusted for age, race, education level, ratio of family income to poverty, energy intake, protein intake, time of venipuncture, serum cotinine, hypertension, diabetes and pubertal status.

1 β: effect sizes.

2 95% CI: 95% confidence interval.

## Discussion

In our cross-sectional study including 1717 male children and adolescents aged 6-19 years, a significant negative association between DII with TT and E2 was observed for male adolescents and remained significant even after adjustment for all potential confounders. Subgroup analysis stratified by BMI suggested that this association was more significant in overweight and obese population, while no significant dependence of BMI on this relationship was observed. In addition, no relationship between DII and sex hormone with statistical significance was detected in male children. Our results indicated that higher consumption of pro-inflammatory diet may contribute to a lower TT and E2 level in male adolescent, the dietary management might be necessary for the development and reproductive health in male adolescents.

To our knowledge, this is the first study assessing the association between the dietary inflammatory potential and sex hormone in male children and adolescents. Many epidemiological studies had found dietary intake could alter sex hormone levels in males. A research based on data from the Massachusetts Male Aging Study, including 1552 men (aged 40-70 years old), suggested fiber intake and age were positively correlated to SHBG levels, while BMI and protein intake were negatively correlated to SHBG levels (44). Carbohydrate-restricted diet (8% carbohydrate) has been proven have no association with total or free testosterone and SHBG (45). Khoo et al. recruited 31 Caucasian male adults with type 2 diabetes mellitus from a community in South Australia and found that diet-induced weight loss (either replacement-based low-calorie diet or low-fat, high-protein, reduced-carbohydrate diet) was significantly correlated with the increase of SHBG, while TT and FT alternation was not statistically significant (46). Moreover, men with high dietary consumption of polyunsaturated fats, monounsaturated fats and polyunsaturated fats combined with CHO (a type of refined carbohydrates) showed drastic decline on net overall testosterone levels (47). Wilson et al. found that TT increased dramatically from weeks 0 to 11 in the ketogenic dieting diet compared with traditional western diet in their study enrolled 25 college-aged men (48). A cross-sectional study conducted by Zhang et al. including 4151 male adults suggested that men with pro-inflammatory diet appeared to have a greater risk of TT decline (28). Using similar NHANES participants, Kuchakulla et al. found higher plant-based diet index score did not have connection with serum testosterone levels (49). Thus, these studies showed that diet could have great impact on sex hormone levels or SHBG. But studies about the relationship between pro-inflammatory diet and sex hormone levels, especially in male children and adolescents has not been reported before. Our study demonstrated that a pro-inflammatory diet may decrease TT and E2 concentrations in male adolescents, while its impact on male children was found not to be statistically significant.

As for the reasons of a non-statistically significant result in male children, firstly, DII calculation was based on 24-h dietary recall data *via* personal interview and children’s food recalls and reports may deviate significantly from the real situation, thus recall bias was inevitable. Secondly, gonadotropins regulate gonadal steroidogenesis, and male children sexual gland are not be well-developed in early stages, which probably means that the dietary influence is hard to detect (50). In addition, the maturation of aromatase activity occurred later in male children (compared with girls), so the impact of pro-inflammatory diet on sex hormone, especially E2, might not be obvious (51). In facts, previous Dietary Intervention Study in Children (DISC) related researches demonstrated that a cholesterol-lowering dietary intervention in male children did not influence serum hormone levels, SHBG, or Tanner stages, which was consistent with our results but inverse from a similar study conducted on girls (52, 53).

Emerging evidences presented those probable mechanisms for pro-inflammation diet induced TT decline might lie on pro-inflammatory markers, including increasing IL-1, IL-6, IL-17 and TNF *etc.* These pro-inflammatory cytokines may lead to hypothalamic inflammation followed by GnRH release decrease, probably resulting in luteinizing hormone (LH) and TT decline (54). Studies conducted by Hales et al. confirmed a close relationship between Leydig cells and interstitial testicular macrophages, and when macrophages were activated and produced inflammatory mediators, the steroidogenesis (mainly testosterone) was greatly inhibited. This inhibition might not only derive from IL-1 and TNF production, but also reactive oxygen species (ROS) secreted by activated macrophages (55). Another study showed that a transient inflammatory response derived from low-dose endotoxin challenge could lead to a decline in serum testosterone without changing LH/FSH concentration, indicating that inflammation might directly impair Leydig cell function (56). Adipose tissue itself was also a major producer of inflammatory cytokines, and obesity was always associated with various degrees of inflammation (57). Visceral adipose tissue (VAT) in male mice, which enriches in proinflammatory genes including Ccl2 etc., recruited more gender-specific Treg cells to abate probable inflammation. And these Treg cells were significantly inhibited without androgen receptor expression. In fact, several epidemiological studies presented positive links among obesity and systemic inflammation with TT decrease, which showed the similar results to our findings (25, 58). A randomized controlled study also revealed testosterone treatment could decrease inflammation and cardiovascular risk (59). As for E2, Schmidt et al. pointed hormonally active testosterone was anti-inflammatory while estrogen was pro-inflammatory, and inflammatory adipose tissue might present higher aromatase function to convert T to E2, which may promote inflammation (60, 61). But aromatase inhibitor to inhibit E2 formation in elderly men with low testosterone levels did not seem to show any connections with inflammation (62). And increased adipose tissue aromatase activity even contributed to adipose tissue inflammation reduction and improved insulin sensitivity in male mice (63). The cause-and-effect relationship between E2 and inflammation still remains controversial and the mechanisms of E2 decline in male adolescent in our study needs further investigation. Our study suggested that a more anti-inflammatory diet could contribute to lessening inflammatory burden and then prevent probable testosterone deficiency in male adolescents.

Limitations of this study cannot be ignored. Firstly, we cannot obtain a clear causal relationship due to the cross-sectional study design. A subsequent large-scale cohort study is necessary to further confirm our results. Secondly, DII calculation was based on 24-h dietary recall, whereas recall bias is inevitable. And it cannot represent daily variability of dietary intake. Thirdly, age span (12-19 years) is a highly variable group in terms of pubertal maturation and thus of sexual steroids levels, although age and pubertal status have been adjusted as covariates, the influence of age span could not be ignored. In addition, serum sex hormone and SHBG concentration was detected at a single time point in NHANES, which cannot represent their diurnal fluctuations. Finally, testosterone levels exhibit a circadian variation with peak levels in the morning and evidence-based guidelines recommend measuring morning TT level (64). Although we included the time of blood sample collection as a covariate and adjusted in the analysis, the bias caused by diurnal fluctuations of TT was inevitable.

## Conclusion

More pro-inflammatory diet was associated with lower TT and E2 level in male adolescent, while no statistically significant association between them was observed in male children. Diet management may be necessary for development and reproductive health benefits in male adolescents. However, more studies are still needed to validate the causal relationship between DII and sex hormones.

## Data Availability Statement

Publicly available datasets were analyzed in this study. This data can be found here: https://www.cdc.gov/nchs/nhanes/.

## Ethics Statement

The studies involving human participants were reviewed and approved by the ethics review board of the NCHS. Written informed consent to participate in this study was provided by the participants’ legal guardian/next of kin.

## Author Contributions

ZQ, Data analysis, Software, Writing-Original draft. NL: Formal analysis, Writing-Original draft. RL, Methodology, Software. LJ, Data analysis. BS, Conceptualization, Funding acquisition, Writing-Reviewing and Editing. All authors contributed to the article and approved the submitted version.

## Funding

This work was supported by the National Natural Science Foundation of China [Grant No. 82000702], the Science and Technology Achievement Transformation Fund of West China Hospital of Sichuan University [Grant No. CGZH19006], the 1.3.5 project for disciplines of excellence from West China Hospital of Sichuan University [Grant No. ZYJC21010], National Clinical Research Center for Geriatrics, West China Hospital, Sichuan University [Grant No. Z2018B10] and Med+ Biomaterial Institute of West China Hospital/West China School of Medicine of Sichuan University [Grant No. ZYME20001].

## Conflict of Interest

The authors declare that the research was conducted in the absence of any commercial or financial relationships that could be construed as a potential conflict of interest.

## Publisher’s Note

All claims expressed in this article are solely those of the authors and do not necessarily represent those of their affiliated organizations, or those of the publisher, the editors and the reviewers. Any product that may be evaluated in this article, or claim that may be made by its manufacturer, is not guaranteed or endorsed by the publisher.
