# Supplementary material for: The Association Between Dietary Inflammatory Potential and Sex Hormones in Male Children and Adolescents Aged 6–19 Years
Source: Front Endocrinol (Lausanne). 2021 Aug 3;12:722941. doi: 10.3389/fendo.2021.722941 (PMC8370775; doi:10.3389/fendo.2021.722941)
Supplement: Supplementary file 1 [file Table_1.docx]

Supplementary Material

**Supplementary Table 1. Baseline characteristics of participants based on DII tertiles, weighted.**

|  | Children (aged 6-11) | | | | Adolescents (aged 12-19) | | | |
| --- | --- | --- | --- | --- | --- | --- | --- | --- |
| DII | Tertile 1  -4.53~-0.57 | Tertile 2  -0.54~1.03 | Tertile 3  1.03~4.08 | P value | Tertile 1  -4.53~-0.51 | Tertile 2  -0.51~1.43 | Tertile 3  1.43~4.58 | P value |
| Age (year) | 8.50 ± 1.67 | 8.55 ± 1.75 | 8.84 ± 1.65 | 0.0767 | 15.27 ± 2.32 | 15.32 ± 2.21 | 15.51 ± 2.16 | 0.3156 |
| Race | | | | | | | | |
| Mexican American | 15.86 | 16.36 | 16.52 | 0.7013 | 16.15 | 16.34 | 16.32 | 0.3416 |
| Other Hispanic | 8.78 | 10.99 | 11.91 |  | 6.32 | 6.95 | 9.14 |  |
| Non-Hispanic White | 56.72 | 51.44 | 47.30 |  | 53.81 | 56.74 | 55.22 |  |
| Non-Hispanic Black | 12.36 | 12.47 | 14.08 |  | 16.08 | 11.72 | 9.64 |  |
| Other Races | 6.29 | 8.73 | 10.19 |  | 7.64 | 8.25 | 9.68 |  |
| Educational level | | | | | | | | |
| Less than high school | 99.85 | 100.00 | 99.77 | 0.7554 | 63.11 | 58.72 | 56.12 | 0.5590 |
| High school or GED | 0 | 0 | 0 |  | 31.97 | 36.68 | 37.50 |  |
| Above high school | 0 | 0 | 0 |  | 4.39 | 3.64 | 5.53 |  |
| Unknow | 0.15 | 0 | 0.23 |  | 0.53 | 0.96 | 0.85 |  |
| PIR (%) | | | | | | | | |
| < 1 | 22.60 | 22.94 | 27.07 | 0.4959 | 24.38 | 18.86 | 21.76 | 0.2549 |
| ≥ 1 | 77.40 | 77.06 | 72.93 |  | 75.62 | 81.14 | 78.24 |  |
| BMI | | | | | | | | |
| Normal | 94.36 | 87.84 | 90.10 | 0.1694 | 72.60 | 65.95 | 64.13 | 0.0970 |
| Overweight | 4.88 | 9.68 | 8.63 |  | 15.94 | 17.81 | 17.67 |  |
| Obese | 0.76 | 2.47 | 1.27 |  | 11.46 | 16.25 | 18.20 |  |
| Time of venipuncture | | | | | | | | |
| Morning | 36.62 | 34.13 | 30.57 | 0.4617 | 48.55 | 41.86 | 42.67 | 0.1115 |
| Afternoon | 41.44 | 38.48 | 44.02 |  | 35.78 | 41.19 | 35.65 |  |
| Evening | 21.94 | 27.40 | 25.41 |  | 15.67 | 16.95 | 21.68 |  |
| Puberty (%) | 6.69 | 5.99 | 6.68 | 0.9353 | 93.85 | 93.43 | 90.73 | 0.2374 |
| Hypertension (%)^1^ | / | / | / | / | 3.61 | 2.89 | 1.97 | 0.7391 |
| Diabetes (%) | 0.27 | 0.36 | 0.24 | 0.9984 | 0.29 | 0.55 | 0.99 | 0.5727 |
| Serum cotinine (ng/mL) | 0.27 ± 0.76 | 0.20 ± 0.60 | 0.31 ± 1.14 | 0.3234 | 16.73 ± 56.30 | 7.29 ± 35.08 | 10.40 ± 48.61 | 0.0358 |
| Energy intake (kcal) | 1470.53 ± 445.67 | 2005.13 ± 573.48 | 2564.44 ± 674.73 | <0.0001 | 1481.82 ± 552.01 | 2197.65 ± 691.08 | 3219.99 ± 1156.28 | <0.0001 |
| Protein intake (g) | 48.98 ± 20.93 | 69.37 ± 21.52 | 96.04 ± 31.57 | <0.0001 | 52.16 ± 23.75 | 84.62 ± 54.90 | 127.83 ± 60.53 | <0.0001 |
| Total testosterone (ng/dl) | 16.65 ± 54.00 | 15.67 ± 50.26 | 19.94 ± 61.45 | 0.6931 | 400.38 ± 222.14 | 363.18 ± 192.71 | 367.68 ± 202.21 | 0.0414 |
| Estradiol (pg/ml) | 2.42 ± 1.38 | 2.50 ± 1.48 | 2.50 ± 1.90 | 0.8421 | 19.63 ± 10.38 | 18.13 ± 10.36 | 18.58 ± 10.05 | 0.1584 |
| SHBG (nmol/l) | 104.44 ± 52.03 | 101.75 ± 51.45 | 102.43 ± 42.48 | 0.8188 | 41.23 ± 24.05 | 39.57 ± 22.72 | 40.79 ± 28.07 | 0.6826 |
| Free androgen index | 0.28 ± 1.00 | 0.35 ± 1.54 | 0.34 ± 1.40 | 0.8153 | 12.51 ± 7.66 | 11.73 ± 7.07 | 12.21 ± 7.57 | 0.4034 |
| Ratio of TT to E2 | 4.53 ± 9.66 | 3.70 ± 7.05 | 4.98 ± 10.04 | 0.2841 | 21.81 ± 11.03 | 21.40 ± 9.57 | 21.29 ± 14.26 | 0.8376 |

Abbreviations: GED, general educational development; PIR, the ratio of family income to poverty; BMI, body mass index; SHBG, sex hormone-binding globulin; TT, total testosterone; E2, estradiol.

^1^Data of hypertension status for participants aged 6-11 years was not available.

**Supplementary Table 2. Subgroup analysis of association between DII and sex hormone stratified by BMI groups in children aged 6-11.**

|  | β^1^ (95% CI^2^), P value | | | | |
| --- | --- | --- | --- | --- | --- |
| DII Tertile | Total testosterone (ng/dl) | Estradiol (pg/ml) | SHBG (nmol/l) | Free androgen index | Ratio of TT to E2 |
| Normal | | | | | |
| Continuous | 0.67 (-1.49, 2.82)  0.5449 | 0.00 (-0.07, 0.07) 0.9783 | -0.05 (-2.97, 2.86) 0.9708 | 0.01 (-0.03, 0.06) 0.5875 | 0.28 (-0.03, 0.60) 0.0783 |
| Tertile 1 | Reference | Reference | Reference | Reference | Reference |
| Tertile 2 | 0.22 (-6.38, 6.83)  0.9470 | 0.09 (-0.12, 0.30) 0.3927 | -3.35 (-12.28, 5.58) 0.4629 | 0.08 (-0.06, 0.22) 0.2685 | -0.51 (-1.48, 0.45) 0.2968 |
| Tertile 3 | 2.09 (-6.09, 10.26)  0.6167 | 0.02 (-0.23, 0.28) 0.8599 | 0.28 (-10.77, 11.32) 0.9611 | 0.06 (-0.11, 0.23) 0.4935 | 1.12 (-0.07, 2.31) 0.0655 |
| Overweight | | | | | |
| Continuous | -0.36 (-10.23, 9.50) 0.9428 | 0.22 (-0.47, 0.92) 0.5334 | -2.46 (-8.02, 3.10) 0.3924 | 0.20 (-0.37, 0.76) 0.5057 | -0.41 (-1.50, 0.68) 0.4694 |
| Tertile 1 | Reference | Reference | Reference | Reference | Reference |
| Tertile 2 | 4.13 (-23.47, 31.74) 0.7712 | 1.28 (-0.64, 3.20) 0.2012 | -1.46 (-17.31, 14.39) 0.8581 | 1.03 (-0.50, 2.56) 0.1980 | -1.67 (-4.48, 1.15) 0.2543 |
| Tertile 3 | 13.13 (-27.63, 53.89) 0.5326 | 1.01 (-1.82, 3.85) 0.4884 | -2.40 (-25.80, 21.00) 0.8422 | 2.08 (-0.18, 4.35) 0.0816 | 2.06 (-2.10, 6.21) 0.3397 |
| Obese^3^ | | | | | |
| Continuous | / | / | / | / | / |
| Tertile 1 | Reference | Reference | Reference | Reference | Reference |
| Tertile 2 | / | / | / | / | / |
| Tertile 3 | / | / | / | / | / |
| P for interaction | 0.7332 | 0.3366 | 0.6172 | 0.1774 | 0.8136 |

The showing results of subgroup analysis was adjusted for adjusted for age, race, education level, ratio of family income to poverty, energy intake, protein intake, time of venipuncture, serum cotinine, hypertension, diabetes and pubertal status；

^1^ β: effect sizes;

^2^ 95% CI: 95% confidence interval;

^3^ The model failed because of the small sample size.
